# Metagenomic and metabolomic analyses reveal the role of gut microbiome-associated metabolites in diarrhea calves

**DOI:** 10.1128/msystems.00582-23

**Published:** 2023-08-24

**Authors:** Zhihai Shi, Yazhou Wang, Xiangzhou Yan, Xiaoya Ma, Anqin Duan, Faiz-ul Hassan, Wenjia Wang, Tingxian Deng

**Affiliations:** 1 Institute of Animal Husbandry and Veterinary Medicine, Henan Academy of Agricultural Sciences, Zhengzhou, Henan, China; 2 Guangxi Provincial Key Laboratory of Buffalo Genetics, Breeding and Reproduction Technology, Buffalo Research Institute, Chinese Academy of Agricultural Sciences, Nanning, Guangxi, China; 3 Institute of Animal and Dairy Sciences, University of Agriculture, Faisalabad, Pakistan; 4 College of Veterinary Medicine, Henan University of Animal Husbandry and Economy, Zhengzhou, Henan, China; The University of Maine, Orono, Maine, USA

**Keywords:** calf diarrhea, Xia-nan cattle, metagenomic analysis, metabolomic analysis, microbiota markers

## Abstract

Calf diarrhea is a multifactorial disease that affects the cattle industry and accounts for more than 50% of calf mortality. Although there is evidence of an association between altered gut microbiota and diarrhea, remarkably little is known about the microbial and metabolic mechanisms underlying the link between gut microbiota dysbiosis and the occurrence of calf diarrhea. Here, we performed fecal metagenomic and metabolomic studies on fecal samples from diarrheic and healthy calves of Xia-nan cattle breed. Results revealed that composition of the gut microbiome and metabolome was remarkably altered in diarrheic calves, and gut microbial alterations were associated with diarrhea and linked to the changes in metabolites. Metabolite profiles showed that diarrheic calves exhibited a marked decrease in some purines (adenosine, adenine, 2'-deoxyguanosine, allantoate, deoxyinosine, and deoxyguanosine) and arachidonic acid (prostaglandin F2α and prostaglandin E2) compared to healthy calves. Purine-producing microbial species, including *Lactiplantibacillus plantarum, Campylobacter coli*, *Treponema porcinum*, *Klebsiella pneumoniae*, and *Phocaeicola coprophilus*, were significantly reduced in diarrheic calves compared to healthy calves, whereas the arachidonic acid-producing species such as *Neisseria gonorrhoeae*, *Staphylococcus aureus*, and *Clostridiales bacterium* exhibited a marked increase. These microbial signatures were closely associated with the metabolic dysbiosis of purine and arachidonic acid in diarrhea calves. Our study showed that gut microbiota-driven metabolic disorders of purine or arachidonic acid were associated with calf diarrhea. The findings prove that altered gut microbiota plays a role in diarrhea pathogenesis and indicate that gut microbiota-targeted therapies could be useful for both prevention and treatment of diarrhea.

**IMPORTANCE:**

Calf diarrhea is of great concern to the global dairy industry as it results in significant economic losses due to lower conception rates, reduced milk production, and early culling. Although there is evidence of an association between altered gut microbiota and diarrhea, remarkably little is known about the microbial and metabolic mechanisms underlying the link between gut microbiota dysbiosis and the occurrence of calf diarrhea. Here, we used fecal metagenomic and metabolomic analyses to demonstrate that gut microbiota-driven metabolic disorders of purine or arachidonic acid were associated with calf diarrhea. These altered gut microbiotas play vital roles in diarrhea pathogenesis and indicate that gut microbiota-targeted therapies could be useful for both prevention and treatment of diarrhea.

## INTRODUCTION

Xia-Nan cattle breed, basically founded by crossing the French Charolais beef cattle male with the Chinese Nanyang cattle female, has important features including fast growth rate and high meat production performance (1). It is well known that calf diarrhea is a multifactorial disease that affects the cattle industry and accounts for more than 50% of all calf deaths (2). To date, calf diarrhea is of great concern to the global cattle industry because of its association with significant economic losses such as reduced milk yield, lower conception rate, and early culling (3 – 5). Changes in microbial communities have long been documented as the primary cause of calf diarrhea (6). Various pathogens, including *Escherichia coli* (7), *Faecalibacterium* (8), *Pseudomonas* spp. (9), *Bacteroidetes* (10), and *Streptococcus* (11)*,* have been recently identified in various combinations from cows diagnosed with calf diarrhea, supporting the bacterial hypothesis of the pathogenesis of these infections.

In order to develop effective treatment strategies for diarrhea, a better understanding of the microbial populations inhabiting the gastrointestinal tract is essential. The gut microbiota is known to play a critical role in maintaining health and disease pathogenesis by influencing both internal and external conditions of the gastrointestinal tract (12). To date, numerous studies have investigated the composition and functional role of the gut bacterial community in animals, such as cattle (13), sheep (14), and humans (15). Although the gut microbiota is known to play a role in the onset of many infectious diseases, including diarrhea, there are few studies on this topic in local Chinese beef. Therefore, it is crucial to understand how the gut microbiome is related to calf diarrhea.

With the development and widespread adoption of next-generation sequencing technology, deep sequencing on samples from specific habitats has become possible enabling research into the relationship between the microbial community and disease. This strategy, including metagenomic sequencing, has been widely used in many animals, such as cattle (16), sheep (17), goat (18), buffalo (19), and humans (20). Metagenomic sequencing is an available method to further investigate into the relationship between the gut bacterial community and diarrhea. The present study aimed to identify the relationship between microbial genes and gut health by characterizing the gut microbiota and its functional diversity in healthy Xia-nan calves and those affected calves with diarrhea.

## MATERIALS AND METHODS

### Animal management and sample collection

Fecal samples were collected from Xia-Nan calves (*n* = 60) housed in the Xia Nan Cattle Modern Agricultural Industrial Park (Beiyang, Henan Province, China). These animals were divided into two groups: diarrheic group (XND, *n* = 30) and healthy group (XNH, *n* = 30). All incidences of calving were classified as normal and occurred between April and June 2022. All calves were subjected to the same management conditions. Briefly, calves were weighed immediately after birth and then housed in individual straw-bedded calf pens (2.5 × 2 m) outside the barn throughout the experimental period (from calf birth until 0.5 mo of age) that separated from their dam. No routine treatments other than navel cleaning were performed. Within 6 h after birth, calves received 4.0 L of their own mother’s colostrum (2.0 L in 2 h, and another 2.0 L in 4–6 h), followed by antibiotic-free processed milk at 10% of body weight twice daily (06:00 and 18:00) during the first 7 days. No waste milk (milk from cows treated with antibiotics or from cows suffering with clinical mastitis) was added to the healthy milk that was given to all calves. All feeding procedures were performed by a professional operator using nipple buckets. Each calf was given free access to clean, fresh water from the third day of life. Free choice hay was administered after the first week of life.

The status and severity of diarrheic calves was assessed using a previously described procedure (21). Overall, fecal scoring was recorded through a standard scoring procedure (0 = normal feces; 1 = semi-formed feces; 2 = loose feces; 3 = watery feces). The detailed information on the sample groups is listed in Table S1. Calves were sampled individually prior to antibiotic administration. Feces were sampled once per calf using the rectal palpation technique while wearing fresh, disposable latex gloves. During sampling, the morphology of the fecal samples was observed and recorded based on criteria described earlier (22), especially the color, consistency, and presence of blood. The fecal sample was placed in the sterile plastic tube, labeled and frozen in liquid nitrogen, and then stored at −80°C until further analysis. Details of sample collection from calves are summarized in Table S1.

### Hematological analysis of calves

Blood samples were collected from 60 calves via the jugular vein at the time of fecal sampling. The samples were collected in twice 5 mL vacuum tubes, one with anticoagulant and the other without. Samples in anticoagulant tubes were used for complete blood count by BC 5000 Vet (Mindray Animal Medical Technology Co., LTD., Shenzhen, China). The analyzed parameters included white blood cells, red blood cells, hemoglobin, hematocrit, mean corpuscular volume, mean corpuscular hemoglobin concentration, and platelets. The samples without anticoagulant were centrifuged at 3,000 g for 15 min at 4°C, and serum biochemistry tests were analyzed using the Catalyst One Chemistry Analyzer (IDEXX Laboratories Inc.), including glucose, blood urea nitrogen (BUN), creatinine, calcium, phosphate, total protein, albumin, and globulin. In addition, 0.5 mL of blood was collected from the jugular vein on the opposite side of each calf using a disposable syringe for the blood parameters test (pH, pCO_2_, and HCO_3_^−^) by Abbott i-STAT 300 analyzer.

### DNA isolation and metagenomic sequencing

DNA was isolated from randomly selected 20 fecal samples (10 samples per group) using the QIAamp Microbiome Kit (Qiagen, Hilden, Germany) according to the manufacturer’s instructions. The concentration and purity of metagenomic DNA was measured using a NanoDrop 2000 spectrophotometer (Thermo Fisher Scientific, Waltham, MA). The quality of metagenomic DNA was confirmed by 1.5% agarose gel electrophoresis. The QIAamp PowerFecal DNA Kit (Qiagen, Maryland, USA) was used to process 200 mg of fecal samples to extract the total DNA according to the manufacturer’s instructions. The TruSeq DNA Sample Prep Kit (Illumina Inc., San Diego, CA, USA) was used to generate paired-end libraries for each sample according to manufacturer guidelines. Covaris M220 (Gene Company Limited, Guangzhou, China) was used to fragment the genomic DNA samples into an average of 350 bp fragments. The resulting fragments were end-repaired, A-tailed, and ligated with the addition of adapters. In addition, the adapters were amplified by PCR, sized-selected, and purified. Finally, a total of 10 XND and 9 XNH samples met the requirements for library construction and were further used for paired-end sequencing on the Illumina NovaSeq System platform (Illumina Inc., San Diego, CA, USA).

### Metagenomic data analysis

Raw sequence data for each sample were filtered to exclude adapter and low-quality sequences using fastp (v0.23.2) software (23). The Burrows-Wheeler Aligner (BWA) (v0.7.17) software (24) was used to filter the reads that correspond to the host bovine genomic DNA sequences (*ARS-UCD1.3*). All clean data were assembled for each sample using the MEGAHIT (v1.2.9) software (25) with the following parameters: -n 0 -q 20. The contigs with lengths greater than 500 bp were used for gene prediction by MetaGeneMark (v3.38) software (26). In addition, the redundant genes were eliminated with 95% identity and 90% coverage of protein sequences by using Cluster Database at High Identity with Tolerance (CD-HIT) (v4.8) software (27). Genes with lengths less than 100 bp and fewer than two mapped reads across all samples were eliminated. The catalog’s non-redundant genes were aligned to the National Center for Biotechnology Information (NCBI) non-redundant (NR) database (version: 2022-12) using Diamond (v2.1.6) software (28) at the threshold of 1E-5. The taxonomic annotation of genes was determined by BASTA (v1.3.2.3) at the thresholds of matching sequence length >25 bp, identity >50%, and annotation shared by at least 60% of the hits based on the least common ancestor algorithms. Genes annotated to Eukaryota (except fungi) were excluded from further analysis.

Microbial alpha diversity parameters including Chao1, Shannon, and Simpson were analyzed using the Mothur (v1.48.0) software (29). Partial least squares discriminant analysis (PLS-DA) was used to assess microbial beta diversity at the gene level. Two multivariate statistical tests [ANalysis Of Similarities (ANOSIM) and Linear discriminant analysis Effect Size (LEfSe) ver1.1.2] were used to assess the differences in species composition and community structure between healthy and diarrheic calves. Gene functional prediction was performed using the Phylogenetic Investigation of Communities by Reconstruction of Unobserved States (PICRUSt2) software (30). Amino acid sequences from the gene catalog were aligned and translated from the gene catalog against the proteins in the Kyoto Encyclopedia of Genes and Genomes (KEGG) database. The R program (ver4.1.3) was used for data visualization and statistical analysis.

### Non-targeted metabolomic analysis of fecal samples

A total of 100 mg stool sample for each individual was placed in an Eppendorf tube for metabolite extractions, followed by the addition of 80% methanol. After vortexing, the samples were incubated on ice for 5 min and centrifuged at 15,000 g for 20 min. The resulting supernatant was used for quality control and analysis. Ultra-high performance liquid chromatography tandem mass spectrometry (UHPLC-MS/MS) analyses were performed using a Vanquish UHPLC system (Thermo Fisher Scientific, Waltham, MA) coupled to a Qrbitrap Q Exactive High-Field (HF) mass spectrometer (Thermo Fisher Scientific, Waltham, MA). Finally, a total of 18 samples met the requirements for non-targeted metabolomic sequencing and were used for further analysis.

Raw data generated by UHPLC-MS/MS were processed using Compound Discoverer 3.1 (Thermo Fisher Scientific, Waltham, MA) to perform peak alignment, peak picking, and quantitation for each metabolite. Peak intensities were then normalized to the total spectral intensity. The normalized data were used to predict the molecular formula based on additive ions, molecular ion peaks, and fragment ions. The peaks were then matched against the mzCloud (https://www.mzcloud.org/), mzVault, and MassList databases to obtain accurate qualitative and relative quantitative results. Subsequently, three databases including KEGG, Human Metabolome Database (HMDB), and LIPID MAPS were used for metabolite annotation. Principal components analysis (PCA) and PLS-DA were performed using MetaX (31) software. Differentially expressed metabolites (DEMs) were identified using univariate data analysis (*t*-test) with the following thresholds: projection (VIP) >1, *P*-value <0.05, and fold change (FC) >1.5 or <0.667. Metabolite function and metabolic pathways for DE metabolites were performed using the KEGG database. The metabolic pathways enriched in DE metabolites were defined as those for which *P* < 0.05 and for which *x*/*n* > *y*/*N*, where *N* refers to the number of metabolites involved in the KEGG pathway; *n*, the number of DEMs in *N*; *y*, the number of metabolites annotated to a KEGG pathway; and *x*, the number of DMs enriched in a KEGG pathway.

### Balance analysis of gut microbial species between diarrheic and healthy calves

To investigate the gut microbial species balance between diarrheic and healthy calves, the balance analysis between divergent gut microbial species and hematological parameters were performed. The *selbal* (32) R package was used to analyze the interaction of gut microbial balance between XND and XNH groups. The function “selbal.cv” in the *selbal* package was used with the parameter “user_numVar” set as 20.

### Correlation analysis of gut microbial species and metabolites

To explore the correlation between blood parameters, gut microbial species, and metabolites, the analysis of sparse partial least squares regression (sPLS) between diarrheic and healthy calves was applied. Briefly, the differential microbiotas between XND and XNH were used based on the results of the LEfSe analysis, and the DEMs were also included. Significantly altered blood parameters (*P* < 0.05) were selected for sPLS analysis. The R package spls (33) was applied to conduct the sPLS analysis. The correlation coefficient was corrected using the “correct.spls” function. The R package pheatmap was used to visualize the relationship between differentially enriched species and metabolites.

To further explore the diagnostic potential of the gut microbiota, we performed the correlation analysis between gut microbial species and selected metabolic pathways using the strategy described by Ye et al. (34). Briefly, the R package PAPi was used to predict the PAPi score of metabolic pathways based on the abundance of the metabolites. Next, the random forest (RF) regression model was used to predict the metabolic pathway with significantly different PAPi scores based on the relative abundance of the gut microbiota species. Additionally, Shapley value of the gut microbiota species in the RF regression model was calculated using R package shapper, which is used to explain the association between the gut species and metabolic pathways. Finally, R package ggplot2 was applied to present the significantly different Shapley values of the gut microbiota species in the metabolic pathway.

### Statistical analysis

All data were presented as mean ± SD. Wilcoxon rank-sum test was used to compare the phenotypic data between the two groups. All statistical tests were two-tailed, and a *P* < 0.05 was considered to be statistically significant.

## RESULTS

### Hematological profile of the study calves

In this study, 30 calves with diarrhea and 30 healthy calves were used. Details regarding their characteristics are presented in Table S2. Birth weight, age, and sex of calves had no significant differences (*P* > 0.05, Wilcoxon rank-sum test) between XND and XNH groups. The analysis of blood parameters showed that only blood pH, HCO_3_^−^, and BUN levels significantly (*P* < 0.05; Wilcoxon rank-sum test) differed between diarrheic and healthy calves. On the contrary, the other blood parameters did not demonstrate significant differences between the two studied groups.

### Metagenomic signature data of diarrheic and healthy calves

To investigate the gut microbiome, 19 available fecal samples were processed and used for metagenomic sequencing. A total of 1.65 GB of raw reads was generated, of which 0.85 and 0.80 GB belonged to XND and XNH groups, respectively (Table S3). After quality filtering and removal of host genome sequences, 1.62 GB of clean reads were retained. Subsequently, 2,267,942 sequences at the scaffold level were generated using the *de novo* assembly (Table S4), with an average N50 length of 7,710.63 bp. Furthermore, a total of 2,570,257 non-redundant genes were predicted, with the total and mean length of 1,710,708,588 bp and 665.58 bp, respectively. The guanine to cytosine percentage of the non-redundant genes was 46.87%. The length of non-redundant genes is shown in Fig. S1A, and the dilution curve of core-pan genes is presented in Fig. S1B. The number of non-redundant genes in XND and XNH is shown in Fig. S1C. Fig. S1D shows that the XND had 1,467,136 unique genes, while the XNH had 392,935 unique genes, and the number of overlapping genes was 710,186.

**Fig 1:**
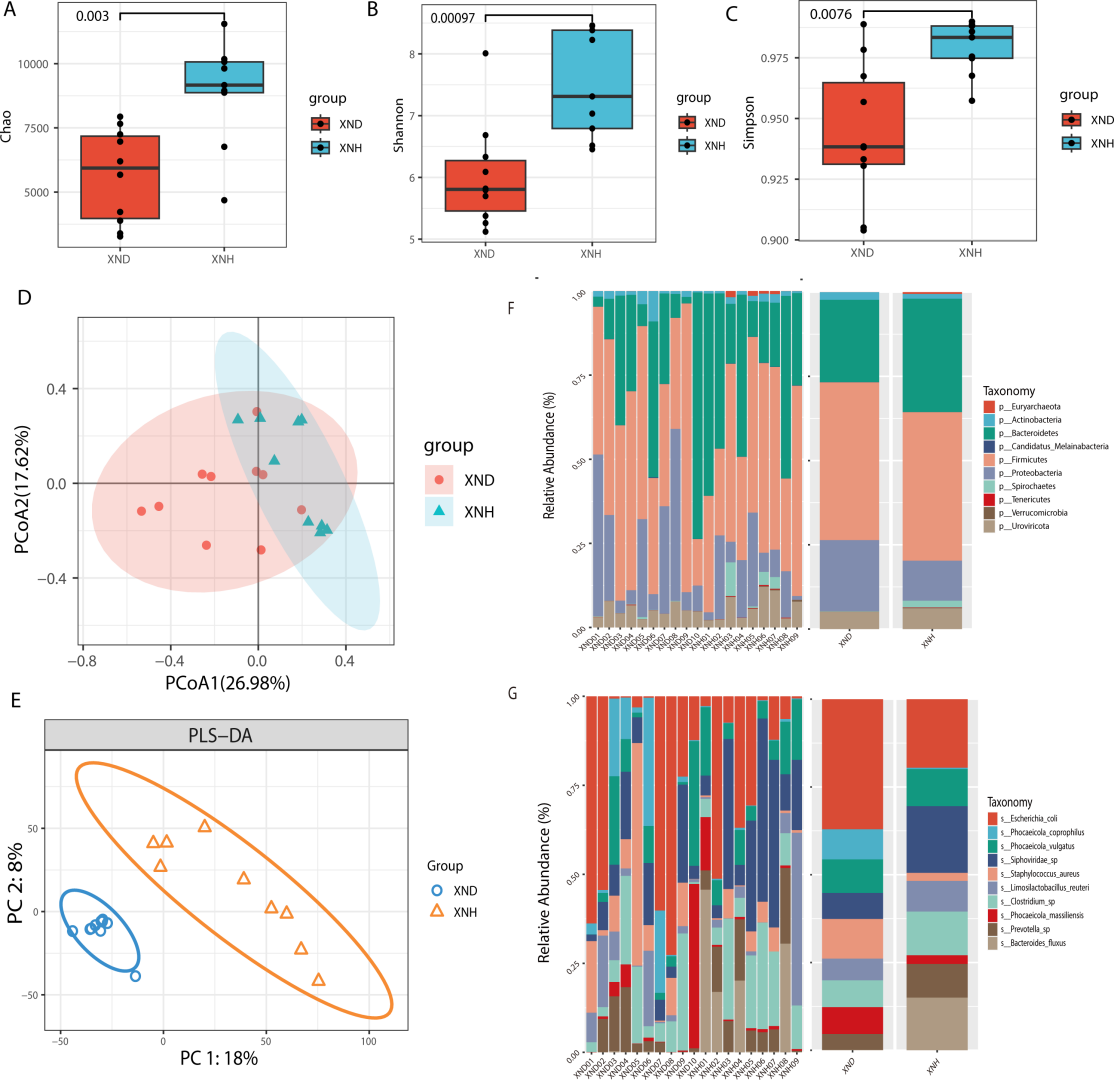
Diversity and composition of gut microbiota between diarrheic and healthy calves. Alpha diversity including Chao1 (**A**), Shannon (**B**), and Simpsons (**C**) was displayed between two groups. Beta diversity including the PCoA (**D**) and PLS-DA (**E**) was analyzed between two groups. PCoA of bacterial community structure based on Buray-Curtis distances. PLS-DA of bacterial community structure based on gene abundance. Histogram showing top 10 relative abundance of phylum (**F**) and species (**G**) among samples or groups.

In the present study, we used three indices (Chao1, Shannon, and Simpson) to evaluate the bacterial alpha diversity between healthy and diarrheic calves. The Chao1 index of the diarrheic group was significantly (*P* < 0.01) lower than that of healthy calves (Fig. 1A), and a similar pattern was exhibited by Shannon (Fig. 1B) and Simpson indices (Fig. 1C). Beta diversities of bacterial communities between healthy and diarrheic calves were calculated and visualized by Principal Coordinate Analysis (PCoA) using the Bray-Curtis distance. The results showed exhibited clear distinction of calves between XND and XNH groups (Fig. 1D). Furthermore, we determined the beta diversity using the PLS-DA which indicated that the gut bacteria in diarrheic calves clustered separately from healthy ones (Fig. 1E).

The top 10 predominant bacterial genera in terms of phylum and species were estimated between XND and XNH calves. At the phylum level, *Firmicutes*, *Bacteroidetes*, *Proteobacteria*, and *Uroviricota* were the major taxa with a relative abundance of more than 0.5% of all bacteria; *Firmicutes* was the predominant phylum, representing more than 43.8% and 34.5% of the total bacteria present in diarrheic and healthy calves, respectively (Fig. 1F). At the species level, a total of 59 bacterial genera were present in diarrheic calves, while the healthy calves had 55 genera with relative abundance greater than 0.5%. Notably, *Escherichia coli* was the most predominant species in the diarrheic and healthy groups representing 29.9% and 18.8% of all bacteria, respectively (Fig. 1G).

Furthermore, we performed bioinformatics analyses regarding the functional annotation of unigenes between the two groups studied. Fig. S2A indicates the unigene number of KEGG, revealing that metabolism had the larger number of unigenes. Among them, carbohydrate metabolism, amino acid metabolism, and metabolism of terpenoids and polyketides were the top 3 unigene numbers for the metabolism pathway. Regarding Gene Ontology (GO) function analyses, Fig. S2B shows that the most abundant GO terms in molecular function, biological process, and cellular component were catalytic activity, metabolic process, and cell, respectively. Regarding the CAZy analyses, three classes of carbohydrate enzymes were enriched, including glycoside hydrolases (GH), glycosyltransferases, and carbohydrate-binding modules (CBMs) as presented in Fig. S2C.

**Fig 2:**
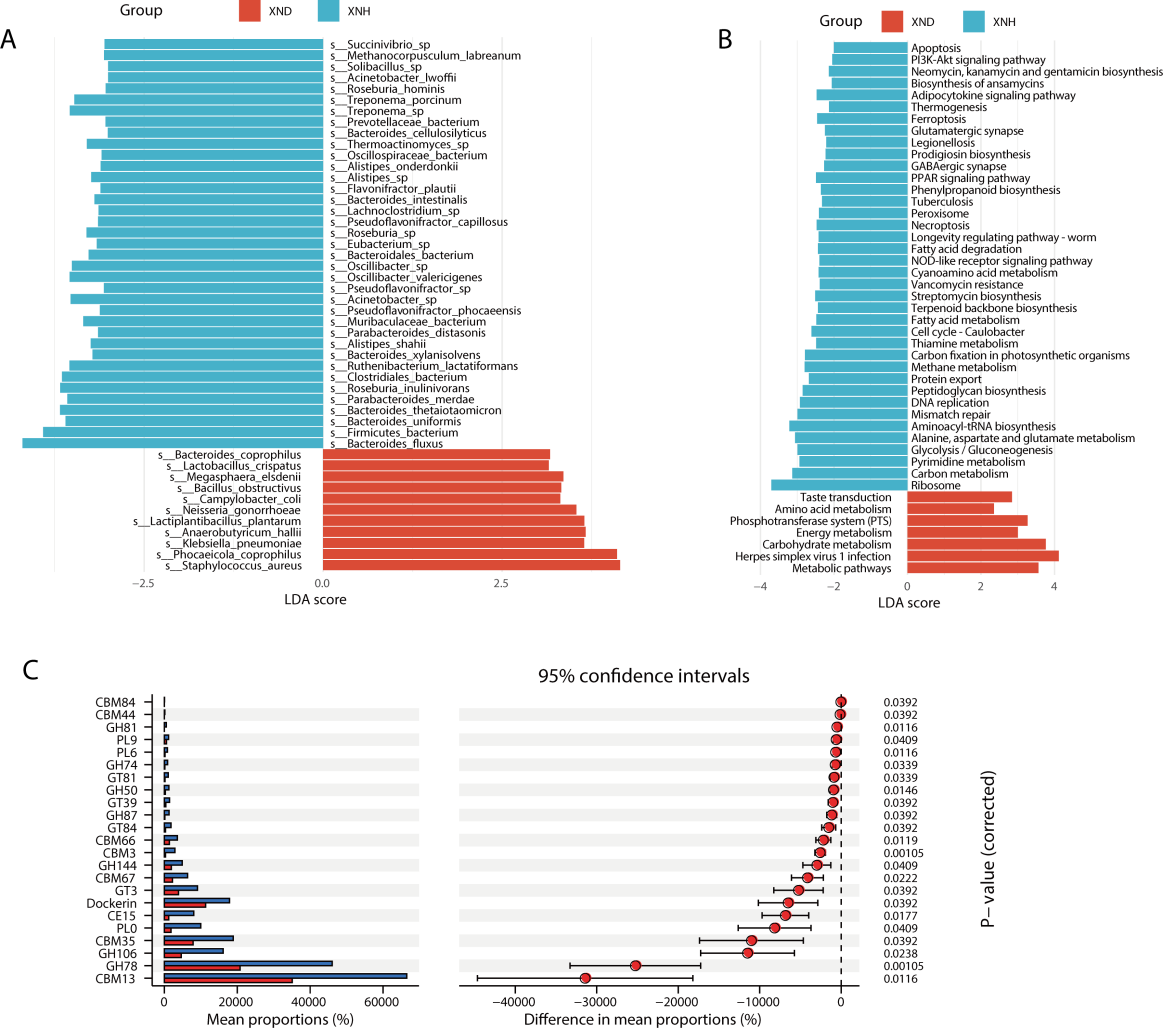
Difference analysis in the gut microbiota between diarrheic and health calves based on metagenomic data. (**A**) LefSe analysis shows the microbiota compositions at the species level between two groups (*P* < 0.05 and LDA > 3). (**B**) LefSe analysis reveals difference of KEGG pathway abundance (*P* < 0.05 and LDA > 2). (**C**) The STAMP analysis of functional differences between the groups of CAZy. The results were filtered using an adjusted *P*-value of 0.05 [Benjamini-Hochberg (BH) correction].

Furthermore, LefSe analysis showed that *S. aureus* [Linear Discriminant Analysis (LDA) = 4.71] had the highest LDA score in the XND group, whereas *Bacteroides fluxus* (LDA = 5.91) had the highest LDA score in the XNH group (all LDA >3, *P* < 0.05; Fig. 2A). Subsequent, KEGG difference analysis showed that the most abundant pathways for diarrheic calves were herpes simplex virus 1 infection, metabolism pathway, and carbohydrate metabolism (Fig. 2B). In contrast, the ribosome, aminoacyl-tRNA biosynthesis, and carbon metabolism were the top 3 abundant pathways in healthy calves. Regarding the CAZy analyses, the functional difference between the XND and XNH groups which were enriched for seven CBMs and seven GHs was performed using the Statistical Analysis of Metagenomic Profiles (STAMP) analysis (Fig. 2C).

### Metabonomics signatures of diarrheic and healthy calves

**Fig 3:**
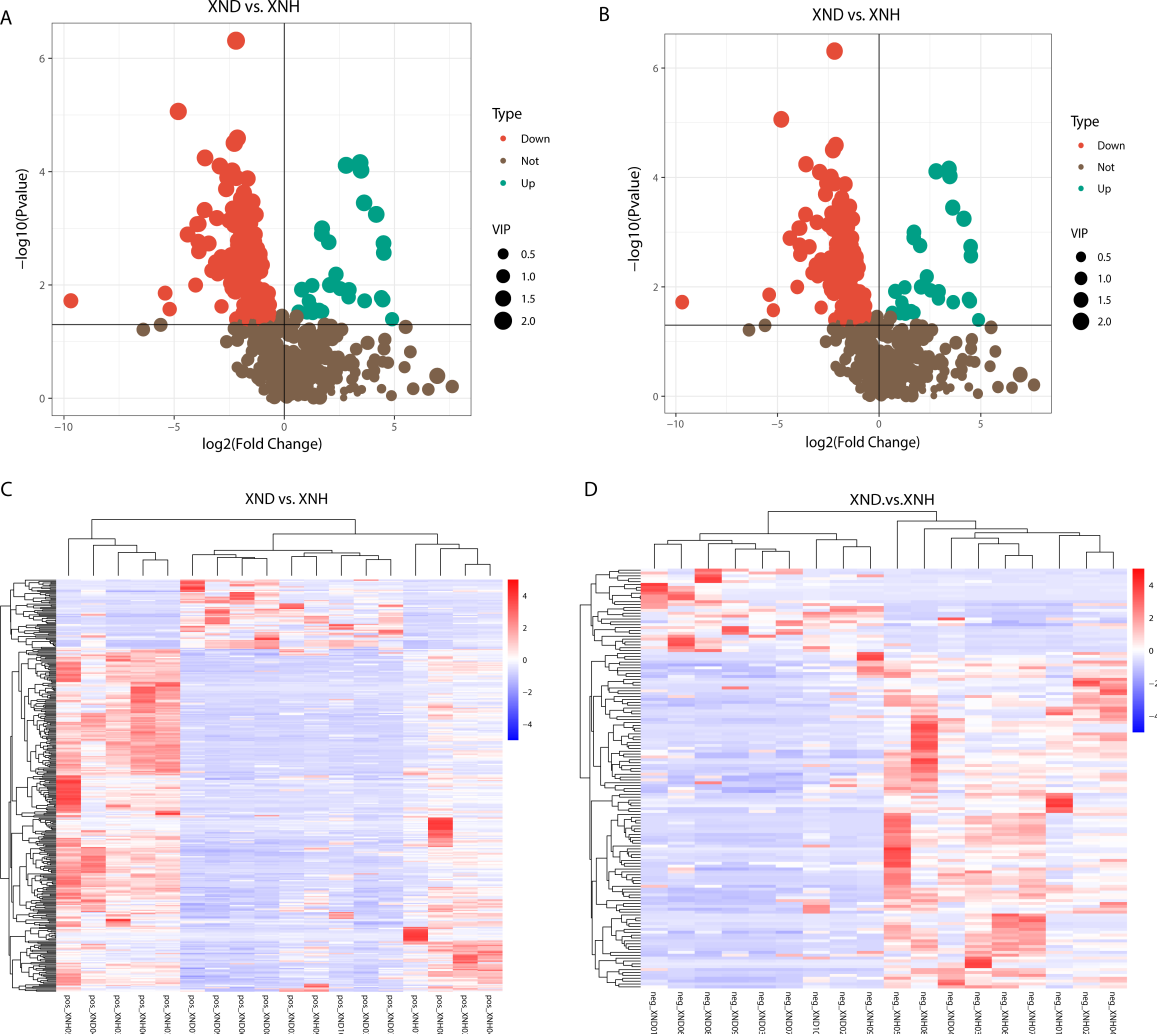
Differentially expressed metabolites between diarrheic and healthy calves. (**A**) Volcano plot indicating upregulated and downregulated metabolites using the positive ion model. (**B**) Volcano plot indicating upregulated and downregulated metabolites using the negative ion model. (**C**) Heat map of DEMs using the positive ion model. (**D**) Heat map of DEMs using the negative ion model.

**Fig 4:**
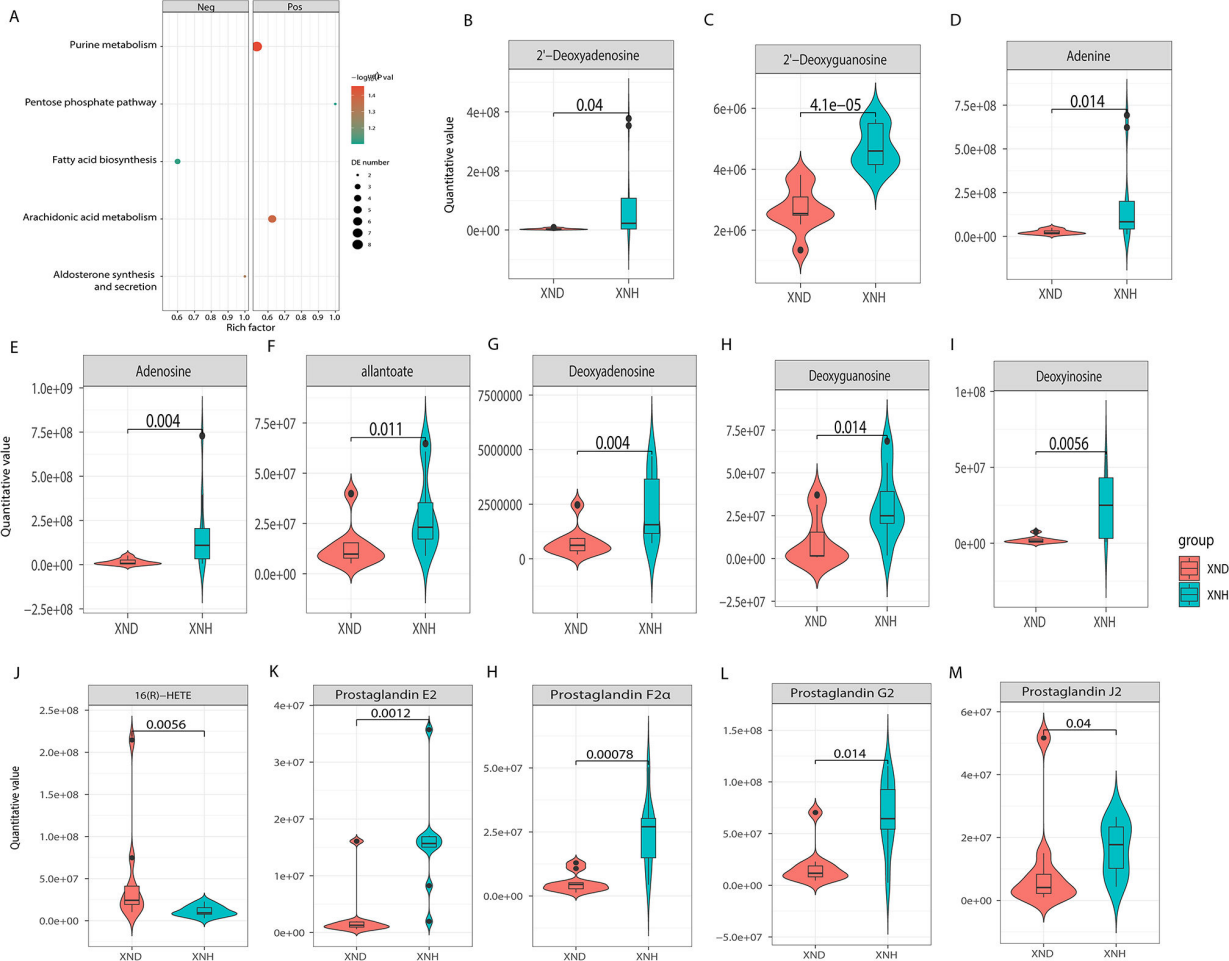
Analyses of differentially expressed metabolites. (**A**) Kyoto Encyclopedia of Genes and Genomes (KEGG) pathway analyses of DE metabolites using the positive or negative ion model. (**B–I**) Violin plot shows quantitative abundance level of the metabolites in purine metabolism. (**J–M**) Violin plot shows quantitative abundance level of the metabolites in arachidonic acid metabolism.

Metagenomic analysis of diarrheic and healthy calves revealed that the microbial metabolism pathways may be crucial in diarrhea. We, therefore, used fecal samples for metabonomic analysis to determine the metabolism function in diarrheic calves. All samples had good quality and system stability based on the results of PCA with positive and negative ions models, respectively (Fig. S3). We identified a total of 362 and 146 DEMs for positive (Fig. 3A) and negative (Fig. 3B) ion models using the significance analysis with projection (VIP) values >1, fold change values >1.5 or fold change values <0.667, and *P*-values <0.05, respectively. The expression pattern of DEMs using positive and negative ion models is shown in a cluster heat map (Fig. 3C and D). Fig. S3 also shows the top 20 of differential fold change in the up- and downregulated DEMs in the positive and negative ion models. Furthermore, KEGG enrichment analysis of DEMs revealed that purine metabolism and arachidonic acid (ARA) metabolism were enriched in the positive model, while no significant signal was observed in the negative model (Fig. 4A), implying that the purine metabolism and ARA metabolism may play important roles in the etiology of diarrhea. In the purine metabolism, a total of eight DEMs displayed a significant (*P* < 0.05) difference between XND and XNH groups (Fig. 4B through I). Additionally, five significant signals for ARA metabolism were observed between XND and XNH groups (*P* < 0.05; Fig. 4J through M).

### Gut microbial signatures significantly correlate with acid-base balance

**Fig 5:**
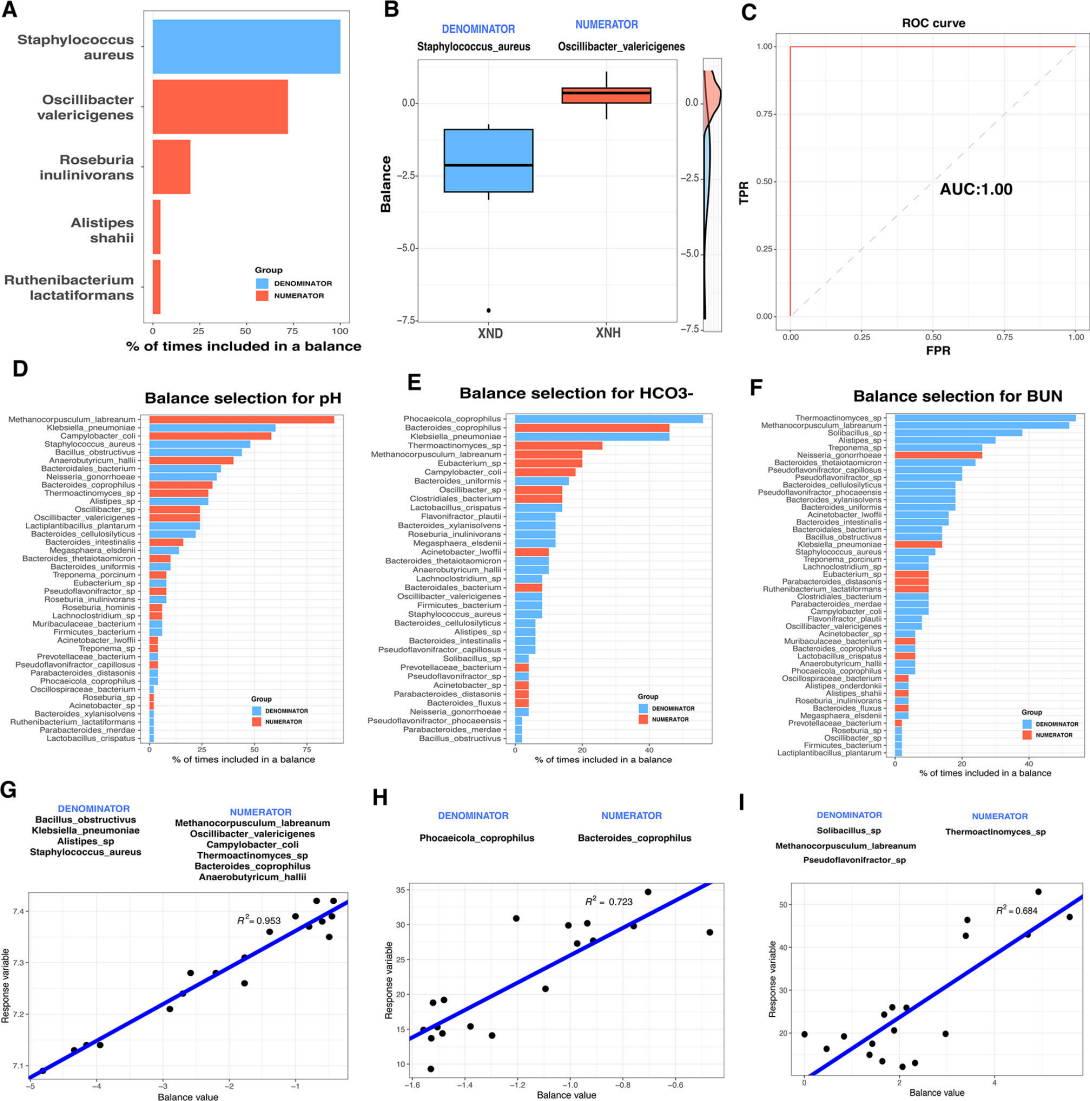
Association of gut microbial species with blood parameters of diarrheic and healthy calves. (**A**) The frequency of gut microbial species selected in the balance of cross-validation process in association with diarrhea. The top-ranked 20 gut microbial species with the highest frequency are shown in the plot. Gut microbial species included in the numerator and the denominator of the balance are presented with blue color and orange color, respectively. (**B**) The two groups of gut microbial signature defining the global balance for diarrhea. (**C**) The apparent AUC value of the balance. (**D–F**) The frequency of gut microbial species selected in the balance of cross-validation process according to the association with blood parameters for pH, HCO_3_^−^, and BUN. (**G–I**) Association of the balance score (x-axis) with blood parameters (y-axis) for pH, HCO_3_^−^, and BUN. *R* ^2^-values were calculated in the regression model.

The balance between metabolically healthy microbiota and dysbiosis has been shown to be critical for maintaining host metabolic homeostasis (35). In this study, selbal analysis was used to determine the balance of gut microbial species between diarrheal and healthy calves. Results showed that *S. aureus* was the most abundant species (over 90%) in the balance of the cross-validation between XND and XNH groups (Fig. 5A). Notably, the samples in the XND group had lower balance scores (Fig. 5B), and the balance showed the best-fit discrimination accuracy [Area Under Curve (AUC) = 1.00, Fig. 5C]. To uncover the association of gut microbial balance with the blood parameters related to acid-base balance, the selbal algorithm was used to search for the microbial signatures. *Methanocorpusculum labreanum* and *Klebsiella pneumoniae* were the top-ranked species associated with the blood pH value (Fig. 5D). For the HCO_3_^−^ indices, *Phocaeicola coprophilus* and *Bacteroides coprophilus* were the top-ranked species (Fig. 5E). *Thermoactinomyces* sp. and *Methanocorpusculum labreanum* were the top-ranked species associated with the BUN (Fig. 5F). The ratio with lower abundance of species such as *Klebsiella pneumoniae* in the denominator to higher abundance of species such as *Methanocorpusculum labreanum* in the numerator was associated with blood pH levels (*R* ^2^ = 0.953; Fig. 5G). Consistently, the ratio of lower abundance *P. coprophilus* to higher abundance *Bacteroides coprophilus* showed a close association with blood HCO_3_^−^ levels (*R* ^2^ = 0.723, Fig. 5H). Likewise, the ratio of lower abundance microbial species (including *Solibacillus* sp., *Methanocorpusculum labreanum,* and *Pseudoflavonifractor* sp.) to high abundance microbial species (*Thermoactinomyces* sp.) was associated with blood BUN (*R* ^2^ = 0.684, Fig. 5I). Thus, the balance of specific microbial species was associated with the acid-base balance of the diarrheic calves.

### Association of gut microbial species with host metabolites and phenotypic traits

**Fig 6:**
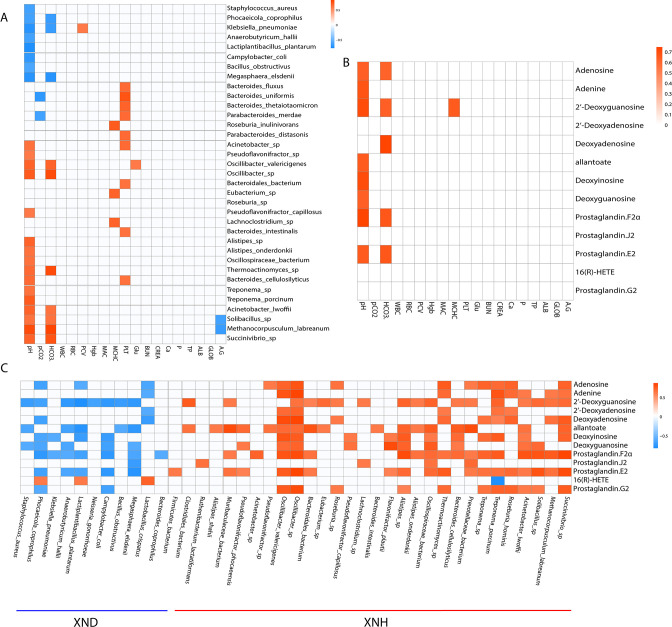
Association of gut microbial species and metabolites with blood parameters between diarrheic and healthy calves. (**A**) Heat map was hierarchically clustered to represent the microbial species-associated blood parameters. (**B**) Heat map was hierarchically clustered to represent the metabolite-associated blood parameters. (**C**) Heat map was hierarchically clustered to represent the microbial species-associated metabolites. The Spearman’s correlation coefficient >0.6 and *P* < 0.05.

To delineate the relationship between metagenomes and metabolomics in diarrheic and healthy calves, we correlated the distinct gut microbiota species and DEMs in purine and ARA metabolism, as well as the blood parameters. The abundance of eight microbial species such as *S. aureus*, *P. coprophilus,* and *Klebsiella pneumoniae* showed a significant (*P* < 0.05) negative association with the blood pH level (Fig. 6A). In contrast, 16 species, including the *Methanocorpusculum labreanum, Solibacillus* sp.*,* and *Pseudoflavonifractor* sp., exhibited a significant (*P* < 0.05) positive association with the blood pH level. Moreover, the abundance of *Methanocorpusculum labreanum* and *Solibacillus* sp. had a significant (*P* < 0.05) positive correlation with the blood HCO_3_^−^ levels, while a significant negative correlation was observed between *P. coprophilus* and *Klebsiella pneumoniae* and blood HCO_3_^−^ levels. Correlation analysis between metabolites and blood parameters further demonstrated that the blood pH level was positively correlated with the adenosine, adenine, 2'-deoxyguanosine, allantoate, deoxyinosine, deoxyguanosine, prostaglandin F2α (PGF2α), and prostaglandin E2 (PGE2) (Fig. 6B). The level of adenosine, 2'-deoxyguanosine, deoxyinosine, PGF2α, and prostaglandin E2 exhibited a positive correlation with blood HCO_3_^−^ level (Fig. 6B). Furthermore, we found that most of the microbiota species enriched in the XND group negatively correlated with the DEMs except for the 16(B)-HETE, while the microbiota species enriched in the XNH group had a positive correlation with DEMs except for the 16(B)-HETE (Fig. 6C). Consequently, our results point out that diarrhea-related alterations of gut microbial species may be involved in blood acid-base balance and disorders in purine or ARA metabolism.

### Diarrhea-associated gut microbial alteration links disordered purine and ARA metabolism

**Fig 7:**
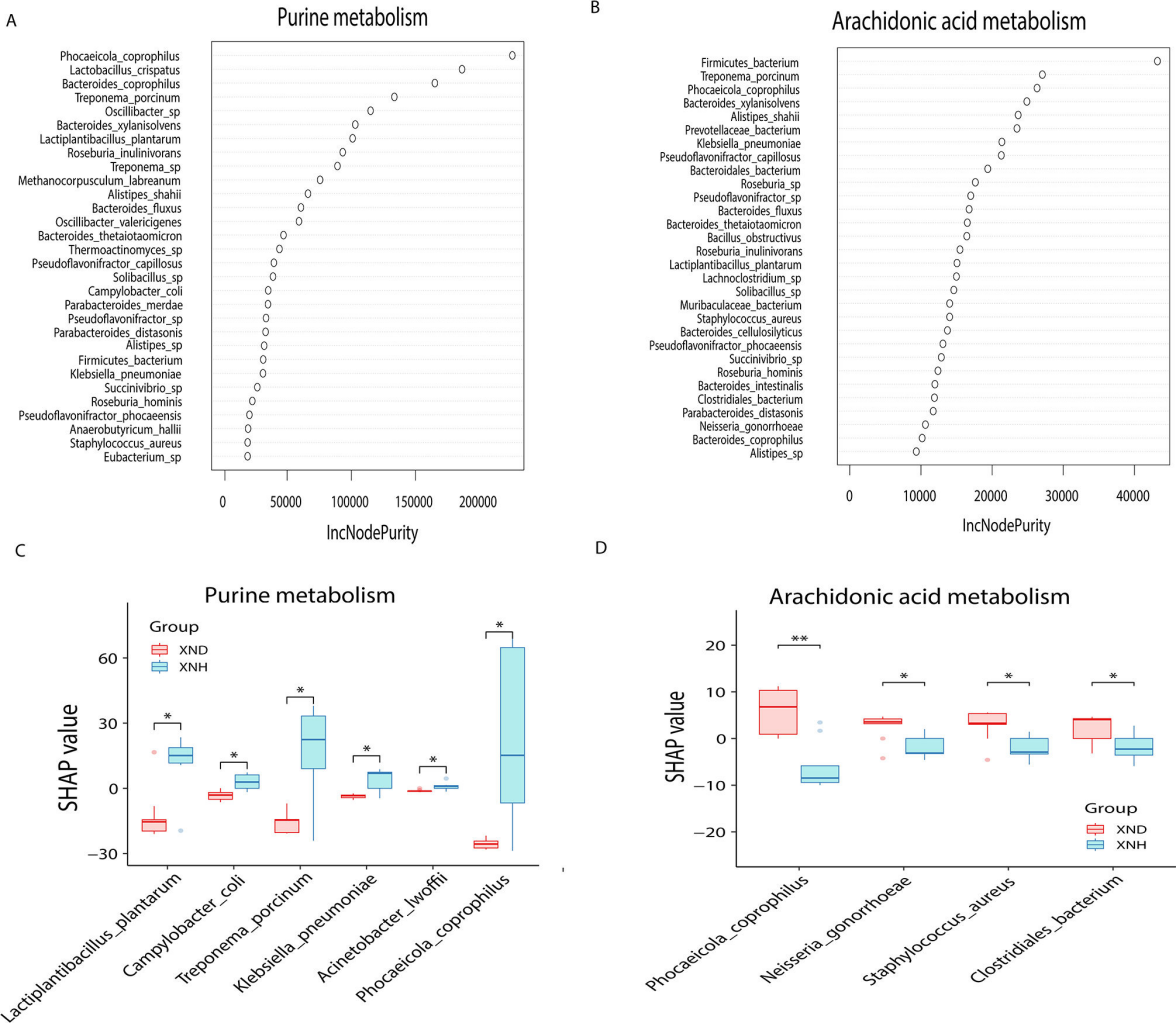
Association between diarrhea-associated gut microbial alteration and disordered purine or arachidonic acid metabolism. (**A**) Random forest model and the gut microbiota species’ importance for purine metabolism. (**B**) Random forest model and the gut microbiota species’ importance for arachidonic acid metabolism. (**C**) Shapley values of gut microbial species with significant changes between XND and XNH groups in association with metabolic signals involved purine metabolism. (**D**) Shapley values of gut microbial species with significant changes between XND and XNH groups in association with metabolic signals involved arachidonic acid metabolism.

Spearman’s correlation analysis revealed that the blood acid-base balance had a strong association with changes in the abundance of gut microbiota or metabolites. To further reveal the association between the diarrhea-associated gut microbial changes and signals in the purine or ARA metabolism, we analyzed the species Shapely values in a random forest regression model. The results demonstrated that *P. coprophilus* had the largest importance value in purine metabolism, followed by *Lactobacillus crispatus*, *Bacteroides coprophillus*, and *Treponema porcinum* (Fig. 7A). For ARA metabolism, we observed that *Firmicutes bacterium* had the largest importance value, followed by the *Treponema porcinum, P. coprophilus,* and *Bacteroides xylanisolvens* (Fig. 7B). Intriguingly, the significantly lower abundance of *Lactiplantibacillus plantarum*, *Campylobacter coli*, *Treponema porcinum*, *Klebsiella pneumoniae, Acinetobacter lwoffii,* and *P. coprophilus* in the diarrheic calves negatively contributed to purine metabolism [false discovery rate (FDR) <0.05, Fig. 7C]. Meanwhile, the higher abundance of *P. coprophilus, Neisseria gonorrhoeae, S. aureus,* and *Clostridiales bacterium* in the diarrheic calves positively contributed to the ARA metabolism (FDR <0.05, Fig. 7D). These results imply that the disordered purine or ARA metabolism in calf diarrhea is significantly influenced by the alteration of gut microbiota species.

## DISCUSSION

Diarrhea, characterized by the inflammation of tissues of gastrointestinal tract, is highly prevalent in cattle. It has been reported that the pathogenesis of diarrhea is complicated and many causative pathogens have been identified, such as *Escherichia coli* (36), *Vibrio parahaemolyticus* (37), *S. aureus* (38), *Salmonella* (39), and *Clostridium perfringens* (40). To date, studies on diarrhea pathogenesis have mainly focused on the composition and function of the bovine gut microbiota (41 – 43). The *Proteobacteria*, *Firmicutes*, and *Bacteroidetes* were three dominant phyla of the gut microbiota in the diarrheic calves, which is quite similar abundance already reported in diarrheic animals (10, 44, 45). However, the microbial and metabolic mechanisms underlying the link between gut microbiota dysbiosis and the occurrence of calf diarrhea remain poorly characterized. In this study, we investigated the functional repertoire of complex bacterial communities in calf diarrhea by integrative analyses of metagenomic and metabolomic analyses. Our findings support the notion that gut microbiota-driven metabolic disorders of purine or ARA were associated with calf diarrhea.

The diversity of gut microbiota is strongly associated with the occurrence of disease (46). In the present study, some bacterial species differed between diarrheic and healthy calves. *Firmicutes*, *Bacteroidetes*, *Proteobacteria*, and *Uroviricota* were four dominant phyla that accounted for a major proportion of gut microbiota in the diarrheic calves, which is quite similar to the abundance previously reported in diarrheic animals (10, 44, 45). More importantly, significant (*P* < 0.05) differences in the abundance of 11 species including *S. aureus*, *P. coprophilus*, *Anaerobutyricum hallii*, *Lactiplantibacillus plantarum*, *Klebsiella pneumoniae*, *Neisseria gonorrhoeae, Megasphaera elsdenii*, *Bacillus obstructivus*, *Campylobacter coli*, *Bacteroides coprophilus*, and *Lactobacillus crispatus* were observed in the guts of diarrheic calves. Among them, seven species, including *S. aureus* (47)*, Lactiplantibacillus plantarum* (48), *Klebsiella pneumoniae* (49), *Megasphaera elsdenii* (50), *Campylobacter coli* (51)*, Bacteroides coprophilus* (52)*,* and *Lactobacillus crispatus* (53)*,* have been reported to be associated with the incidence of diarrhea. The obtained results indicate that these altered bacterial species may be essential for the host adaptation to diarrhea or environments.

ARA is an integral component of the biological cell membrane and plays a vital role in various physiological functions such as skeletal muscle (54), immune system (55), and ionic balance (56). Evidence suggests that prostaglandins may play a role in the pathogenesis of diarrhea and shock associated with bacterial infections (57, 58). Compelling evidence has showed that PGE2 induces diarrhea and reduces water absorption by the digestive tract (59), while PGF2α may cause increased smooth muscle tone linked to diarrhea (60). Prostaglandin G2 (PGG2) is formed from ARA and is rapidly converted into prostaglandin H2 which is a precursor for many other biologically significant molecules including PGE2 and PGF2α (61). In the present study, our metabolomics data revealed that the PGE2 and PGF2α, as well as PGG2 and prostaglandins J2 in fecal samples from diarrheic calves were significantly (*P* < 0.05) lower than in healthy calves. Furthermore, the integrated analysis showed a significant negative correlation between altered gut microbial species (*P. coprophilus, Lactiplantibacillus plantarum, Anaerobutyricum hallii, Klebsiella pneumoniae,* and *Campylobacter coli*) and ARA metabolism. Jung et al. (62) have shown that the *Lactiplantibacillus plantarum* can improve bowel symptoms, stool consistency, quality of life, beneficial microbiota, and overall intestinal health. Both *Klebsiella pneumoniae* (63) and *Campylobacter coli* (64) have been reported to be associated with diarrhea. Therefore, these findings suggested that these diarrhea-associated gut species might contribute to ARA dysfunction.

Uric acid (UA) is the end product of purine metabolism that contributes to metabolic disorders (65). Metabolic disorders are closely associated with intestinal diseases, and the link is usually established with the gut microbiota as a node (66). In this study, the metabolomics data revealed that diarrheic calves had significantly (*P* < 0.05) decreased fecal adenine, adenosine, allantoate, deoxyadenosine, deoxyguanosine, deoxvinosine, 2'-deoxyguanosine, and 2'-deoxyadenosine. This means that the diarrheic calves had an abundance of purine and its derivatives. Of note, correlation analyses revealed that adenine, adenosine, allantoate, 2'-deoxyguanosine, deoxyadenosine, and deoxyguanosine were positively (*P* < 0.05) associated with blood pH and HCO_3_^−^ levels. Allantoic acid (allantoate) was shown to be a degradative product of UA (67). In addition, the diarrheic calves had lower (*P* < 0.05) blood pH and HCO_3_^−^ levels than the healthy calves. Thus, we concluded that these purines and their derivatives may contribute to the metabolic disturbances that contribute to the occurrence of diarrhea. Moreover, an integrated analysis confirmed a significant negative correlation between altered gut microbial species (*S. aureus, P. coprophilus, Lactiplantibacillus plantarum, Neisseria gonorrhoea, Anaerobutyricum hallii, Klebsiella pneumoniae, Megasphaera elsdenii,* and *Campylobacter coli*) and purine metabolism. Goncheva et al. (68) have reported an association between purine metabolism and virulence in *S. aureus*. Morse and Bartenstein (69) found that purine metabolism plays a critical role in the growth of *Neisseria gonorrhoeae*. Therefore, these findings suggest a link between the diarrhea-related altered gut microbiome associated with purine metabolic disorders.

Furthermore, it has been shown that several key blood parameters such as pH, HCO_3_^−^, and BUN are the diagnostic hallmarks of calf diarrhea (70, 71). Here, we observed that these blood parameters had significantly varied between the health and diarrhea groups. Additionally, the pleiotropic effects of the gut microbiota on host metabolism are mediated in large part by the microbial metabolites produced by the gut microbiota (72). Gut microbiota balance has been strongly associated with gastrointestinal disease (73), and is critical for maintaining host metabolic homeostasis (74). In the present study, we found that some gut microbiota species differed between diarrhea and healthy calves. Blood pH levels had a strong positive correlation (*R* ^2^ = 0.953) with the ratio of lower abundance of some microbial species such as *Klebsiella pneumoniae* to the higher abundance of species such as *Methanocorpusculum labreanum*. Evidence showed that the *Klebsiella pneumoniae* tolerated a pH concentration from 7 pH to 10 pH, indicating its tolerance to both neutral and alkaline conditions (75), while the *Methanocorpusculum labreanum* grew over a narrow pH range, with rapid growth near pH 7 (76). This means that the imbalance of these species contributes to the regulation of blood pH level. In addition, our findings showed that the ratio of lower abundance of *P. coprophilus* to higher abundance of *Bacteroides coprophilus* showed a close association with blood HCO_3_^−^ (*R* ^2^ = 0.723). Previous studies have confirmed that *Bacteroides coprophilus* could regulate the abundance of intestinal short-chain fatty acids (77), while the luminal short-chain fatty acid could stimulate the bicarbonate secretion (78). This implies that dysfunction of these species was related to blood HCO_3_^−^ levels. It is well documented that both pH and HCO_3_^−^ are important blood parameters that characterize the acid-base balance (79). Compelling evidence showed that acid-base balance in calves was associated with diarrhea (80, 81). Thus, our results reveal that the imbalance of the gut microbiota may be associated with the occurrence of diarrhea in calves by controlling the acid-base balance.

### Conclusion

The integrative analysis of metagenomic and metabolomic data in the present study identified the disturbance of purine or ARA metabolism and acid-base imbalance as gut microbiota-derived multiple hits associated with calf diarrhea. These findings may provide coherent insights to explain how interactions between microbes and metabolites affect the pathogenesis of calf diarrhea. The results obtained also highlight the potential of targeting the gut microbiota and microbial metabolites as potential markers for innovative therapeutic approaches to treat diarrhea.

## Data Availability

Metagenomic sequencing data are available on NCBI SRA with an accession number of PRJNA956982. Metabolite sequencing data are available on China National GeneBank Database (CNGBdb) with an accession number of CNP0004562.

## SUPPLEMENTAL MATERIAL

Figure S1 msystems.00582-23-s0001.docx Statistics information of *de novo* assembly for metagenome sequencing.

Figure S2 msystems.00582-23-s0002.docx Functional annotation of ungenes from metagenome sequencing.

Figure S3 msystems.00582-23-s0003.docx PCA and different expressed analyses of metabolites.

Table S1 msystems.00582-23-s0004.xlsx Information of all studied diarrhea calves in the present study.

Table S2 msystems.00582-23-s0005.xlsx Demographic and blood parameters of calves with and without diarrhea.

Table S3 msystems.00582-23-s0006.xlsx Statistics information of metagenome sequencing for 19 samples.

Table S4 msystems.00582-23-s0007.xlsx Assembly analysis for gut metagenome in the diarrhea and healthy feces.

ASM does not own the copyrights to Supplemental Material that may be linked to, or accessed through, an article. The authors have granted ASM a non-exclusive, world-wide license to publish the Supplemental Material files. Please contact the corresponding author directly for reuse.
