# Supplementary material for: Metagenomic and metabolomic analyses reveal the role of gut microbiome-associated metabolites in diarrhea calves
Source: mSystems. 2023 Aug 24;8(5):e00582-23. doi: 10.1128/msystems.00582-23 (PMC10654109; doi:10.1128/msystems.00582-23)
Supplement: Figure S1 — Statistics information of de novo assembly for metagenome sequencing. [file msystems.00582-23-s0001.docx]

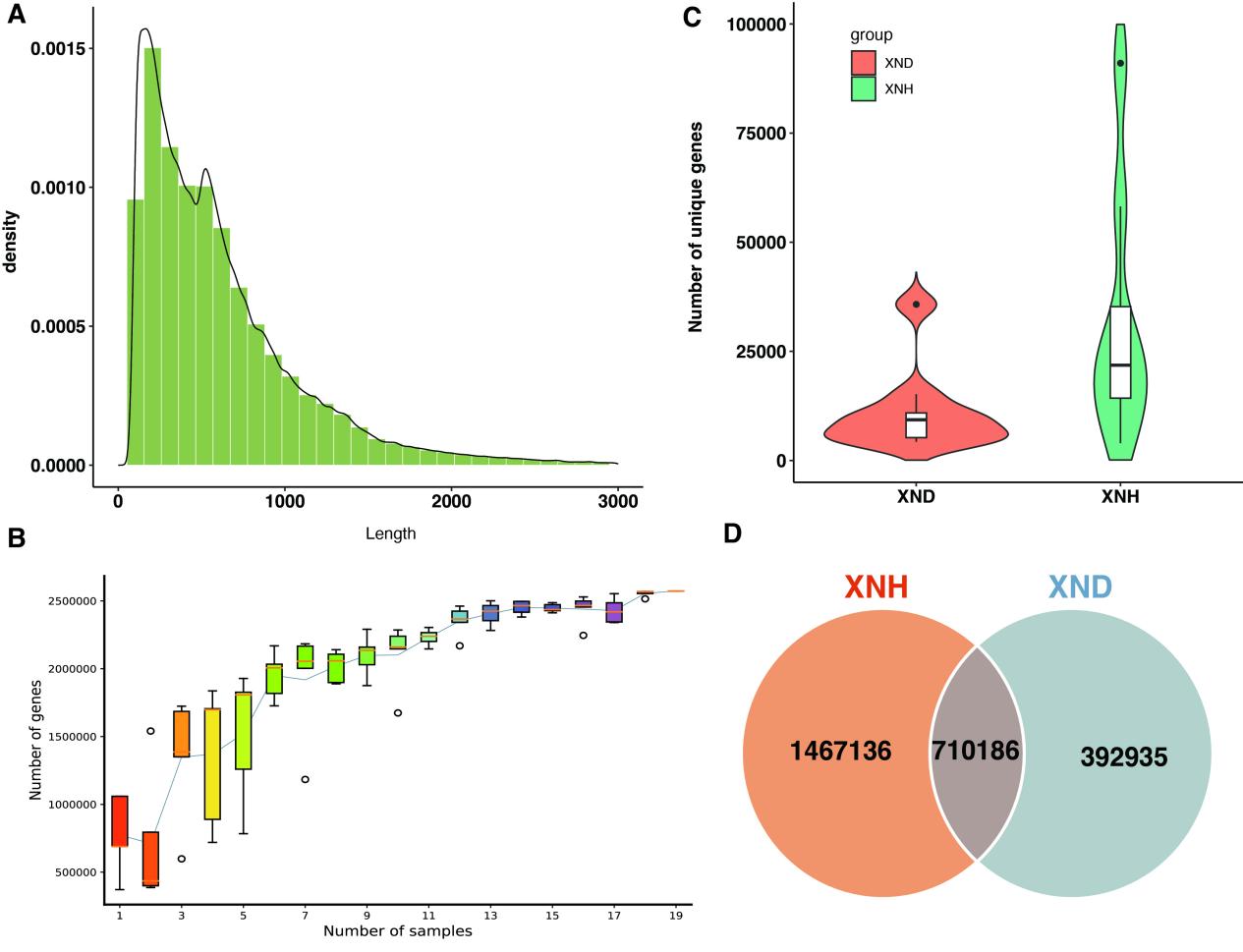


**Fig. S1.Statistics information of *de novo* assembly for metagenome sequencing.** (A)Length distributio of non-redundant genes.(B) The dilution curve of core-pan genes. (C) the number of unigene genes between XND and XNH group. (D) Venn analysi reveals the shared number of unigene genes.
