# Supplementary material for: Metagenomic and metabolomic analyses reveal the role of gut microbiome-associated metabolites in diarrhea calves
Source: mSystems. 2023 Aug 24;8(5):e00582-23. doi: 10.1128/msystems.00582-23 (PMC10654109; doi:10.1128/msystems.00582-23)
Supplement: Figure S2 — Functional annotation of ungenes from metagenome sequencing. [file msystems.00582-23-s0002.docx]

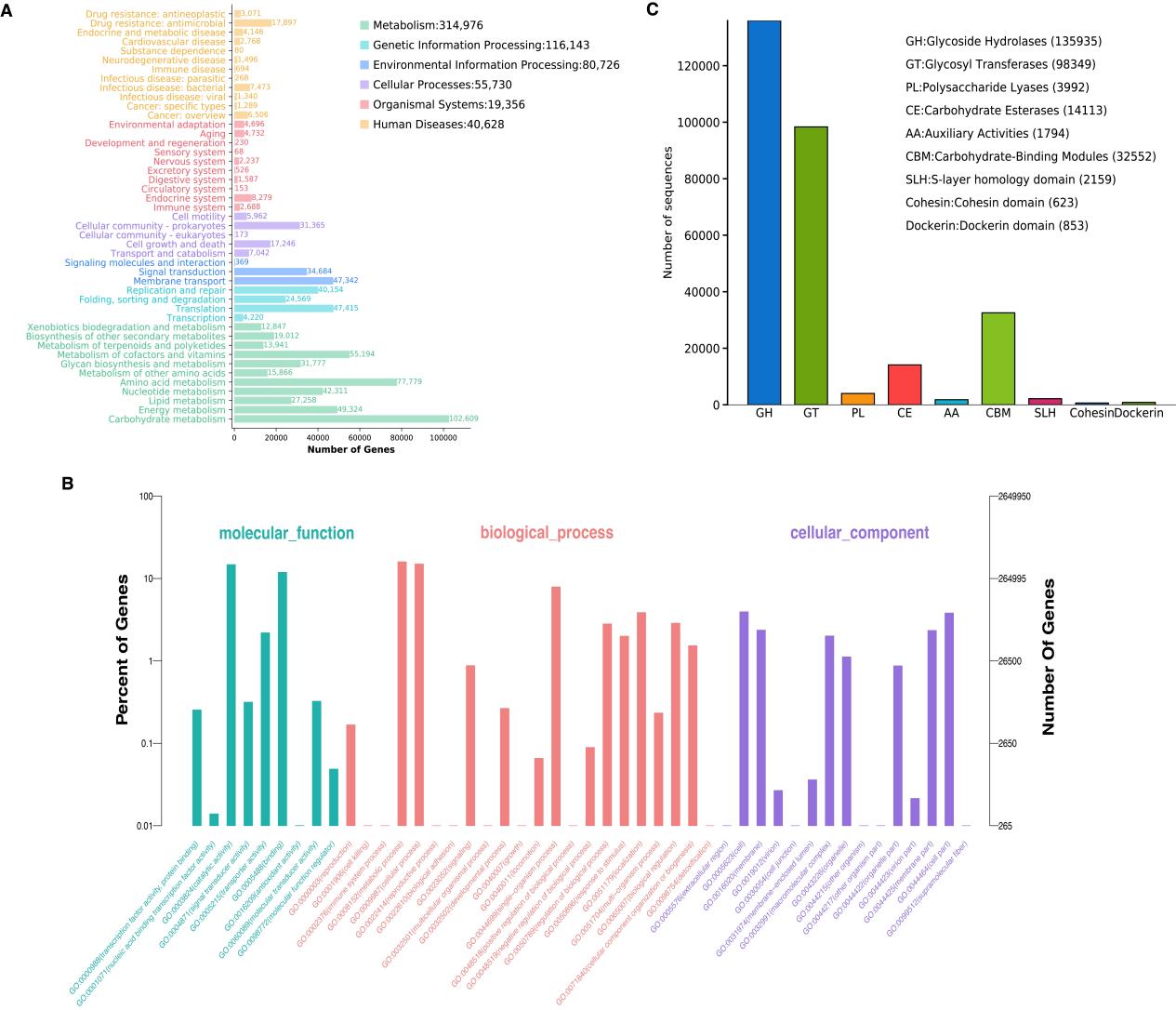


**Fig. S2. Functional annotation of ungenes from metagenome sequencing** (A) Funcational annotation of unigenes. (B) the most abundant GO enrichment analysis of unigenes. (C) the CAZy annotation analyses of unigenes.
