# Supplementary material for: Metagenomic and metabolomic analyses reveal the role of gut microbiome-associated metabolites in diarrhea calves
Source: mSystems. 2023 Aug 24;8(5):e00582-23. doi: 10.1128/msystems.00582-23 (PMC10654109; doi:10.1128/msystems.00582-23)
Supplement: Figure S3 — PCA and different expressed analyses of metabolites. [file msystems.00582-23-s0003.docx]

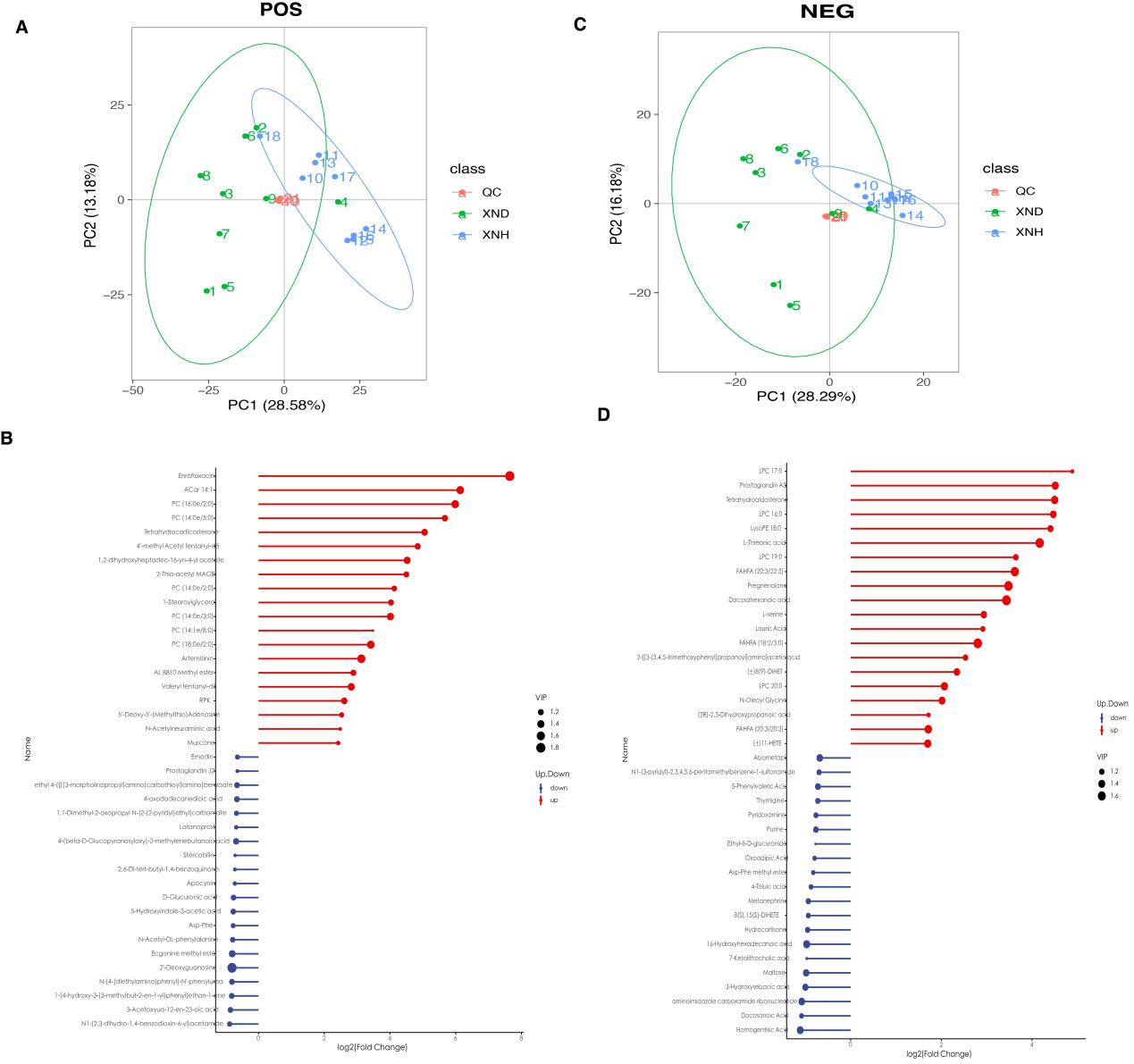


**Figure S3. PCA and different expressed analyses of metabolites** (A)PCA analysis of metabolites for POS. (B)the stick plot displays the different expressed metabolites for POS. (C)PCA analysis of different expressed metabolites for NEG.(D)the stick plot displays the different expressed metabolites for NEG.
